# The Membrane Proteins Involved in Virulence of *Cronobacter sakazakii* Virulent G362 and Attenuated L3101 Isolates

**DOI:** 10.3389/fmicb.2015.01238

**Published:** 2015-11-09

**Authors:** YingWang Ye, Jina Gao, Rui Jiao, Hui Li, Qingping Wu, Jumei Zhang, Xian Zhong

**Affiliations:** ^1^School of Biotechnology and Food Engineering, Hefei University of TechnologyHefei, China; ^2^State Key Laboratory of Applied Microbiology Southern ChinaGuangzhou, China; ^3^Guangdong Provincial Key Laboratory of Microbiology Culture Collection and ApplicationGuangzhou, China; ^4^Guangdong Institute of MicrobiologyGuangzhou, China

**Keywords:** membranous proteins, *Cronobacter sakazakii*, virulence factors, two-dimensional gel electrophoresis (2-DE)

## Abstract

*Cronobacter sakazakii* is an opportunistic foodborne pathogen and the virulence differences were previously documented. However, information about membranous proteins involved in virulence differences was not available. In this study, virulent characterization such as biofilm formation and flagella motility between virulent *C. sakazakii* isolate G362 and attenuated L3101 were determined. Then, two-dimensional gel electrophoresis (2-DE) technology was used to preliminarily reveal differential expression of membranous proteins between G362 and L3101. On the mass spectrometry (MS) analysis and MASCOT research results, fourteen proteins with differential expression were successfully identified. At the threshold of twofold changes, five out of eight membranous proteins were up-regulated in G362. Using RT-PCR, the expression abundance of the protein (*enzV, ompX, lptE, pstB*, and *OsmY*) genes at mRNA levels was consistent with the results by 2-DE method. The findings presented here provided novel information and valuable knowledge for revealing pathogenic mechanism of *C. sakazakii*.

## Introduction

The genus of *Cronobacter* is a group of opportunistic pathogens linked with life-threatening infections in neonates (Holý and Forsythe, 2014). *Cronobacter* sp. was isolated from various kinds of samples including food, soil, environments, clinical samples (Gurtler et al., 2005). In addition, these infections often occurred among poor immunity or low-birth weight newborn through consumption of contaminated powdered infant formula (Muytjens et al., 1983; Tall et al., 2014). The *Cronobacter* genus consists of *Cronobacter sakazakii*, *C. malonaticus*, *C. turicensis*, *C. muytjensii*, *C. condiment*, *C. universalis*, and *C. dublinensis* (Joseph et al., 2012a,b). Furthermore, *C. sakazakii* is the predominant species within *Cronobacter* isolated from powdered milk (Xu et al., 2014).

Membranous proteins were required for adhesion, invasion, and the release of toxin of pathogens, and further played important roles on host-pathogen interaction or host infections in foodborne pathogens (Allaoui et al., 1993; Monteville et al., 2003; Ellis and Kuehn, 2010). Transmembrane signal proteins of two-component regulatory system in gram-negative pathogens contributed to immune escape or biofilm formation (Krachler et al., 2011). In addition, outer membrane proteins A (OmpA) and X (OmpX) in *C. sakazakii* played critical roles in adhesion to and invading human cells (Mohan Nair and Venkitanarayanan, 2007; Mohan Nair et al., 2009; Kim et al., 2010). Kothary et al. (2007) reported that *zpx* gene encoding the cell-bound zinc-containing metalloprotease might play important roles in dissemination of *Cronobacter* into the systemic circulation. However, little information about membranous proteins involved in virulence differences among *C. sakazakii* isolates is available.

To determine the potential virulence factors of pathogenic bacteria, the use of global techniques such as proteomics was widely applied in exploring different factors between virulent and attenuated bacteria (Srinivasa Rao et al., 2004; Hecker et al., 2010; Niemann et al., 2011).

In this study, the virulent characterization including biofilm formation, flagella motility, and O serotypes between G362 and L3101 was determined. Then, the 2-DE technology coupled with mass spectrometry was applied to identify global molecular information about differentially expressed membranous proteins. Finally, Real-time PCR was used to further validate the expression abundance of these novel membranous protein genes between G362 and L3101.

## Materials and Methods

### *Cronobacter sakazakii* Isolates

The virulence differences between *C. sakazakii* G362 and L3101 from food samples were previously determined through intraperitoneal injection and histopathologic analysis (small intestine, kidney, and liver; Ye et al., 2015). The significant morphological change in small intestine, liver, and kidney further reveals the virulence differences between G362 and L3101.

### Different Phenotypic Characterization between G362 and L3101

The biofilm formation between G362 and L3101 was determined using crystal violet staining (CVS) method as described by Hochbaum et al. (2011). The O serotypes were detected as described by Sun et al. (2012). In addition, the primers, PCR conditions, and PCR mixtures also referred to the description by Sun et al. (2012).

In addition, the motility of two isolates (G362 and L3101) was also determined. Media consisting of 10 g/L tryptose, 5 g/L NaCl, and 1.5 g/L agarose was used to test the swimming motility. Swimming plate was inoculated with *C. sakazakii* isolates from an overnight culture of TSA agar using a sterile toothpick and incubated at 37°C for 8 h. Media (8 g/L nutrient broth, 5 g/L D-glucose, and 5 g/L Difco bacto-agar) was made to determine the swarming motility. Swarming plate was inoculated with *C. sakazakii* isolates from swimming plate using a sterile toothpick and incubated at 37°C for 12 h. The diameter of motility at the agar dish interface was measured in triplicate.

### Membranous Proteins Extraction and Quantification

Two *C. sakazakii* isolates (G362 and L3101) were inoculated into tryptic soy broth (TSB, Huankai, Guangzhou) for incubation at 37°C for 16 h. Two grams (wet weight) of G362 and L3101 were suspended in 5 ml of phosphate buffer saline (PBS, pH7.4) at 4°C. Then the cell suspension was sonicated in an ice bath (SONICS VC-505). Following the sonication process, Triton X-114 was applied for membranous proteins extraction using the method described by González de la Vara and Alfaro (2009). Then, two different phase collections and the insoluble pellet were performed to analyze SOD activity (Huang et al., 2006). Acetone precipitation was employed to remove the excess salts in the aqueous phase collection and detergent phase collection respectively. After lysis buffer (5 M urea, 2 M thiourea, 2% SB3-10, 2% CHAPS, 65 mM DTT, 40 mM Tris; Bio-Rad, USA) dissolution and centrifuged at 18, 000 ×*g* for 1 h at 4°C, membrane proteins supernatant was stored at -80°C for use. Proteins qualification was measured by the bicinchoninic acid (BCA) assay as described by Krieg et al. (2005).

### 2-DE Analysis

For each sample, 100 μl (8 μg/μl) of membrane proteins extract, diluted with 200 μl of rehydration buffer (7 M urea, 2 M thiourea, 1% ASB-14, 65 mM DTT, 0.2% Bio-Lyte (w/v), 40 mM Tris, 0.001% Bromophenol blue; Bio-Rad, USA), was applied to a reswelling cassette with 17 cm immobiline dry strip (pH 3-10 NL, Bio-Rad, USA). Rehydration was allowed to proceed at 20°C to 16 h under silicone oil. The isoelectric focusing (IEF) was performed using the PROTEAN IEF system (Bio-Rad, USA). Each IPG strip current limit during IEF was set 50 μA at 20°C. The focusing was conducted in next five steps: (1) constant voltage at 250 V for 1 h; (2) constant voltage at 500 V for 30 min; (3) constant voltage at 1000 V for 2 h; (4) under linear ramping mode voltage increased from 1000 to 10, 000 V in 5 h; (5) at last constant voltage at 10, 000 V until the total volt-hours reached 68,000 V-h. The following steps including equilibration, SDS-PAGE electrophoresis, coomassie brilliant blue stain (CBB-stained), gel analysis, and subsequent MS analysis were performed as described by Cordwell et al. (2008) with modification. In brief, spots in gels between isolates corresponding to the same protein identifications were detected using PD-Quest (Bio-Rad) and the relative spot intensities calculated. Significantly changed spots were selected by rate increased/decreased twofold. Then Protein spots of interest were subjected to in-gel digestion, and then cut from the gels for mass spectrometric analysis. Protein identities were based on a combination of peptide fingerprint and MS/MS spectra. MS and MS/MS data were searched using MASCOT version 1.9.05 (Matrix Science) as search engine against the NCBI database. *C. sakazakii* species database was defined as a matching species. For each isolate sample, the 2-D gels in triplicate were analyzed.

### Expression Abundance of Membranous Protein Genes by Real Time PCR

The overnight culture (1.0 ml) of *C. sakazakii* isolates was subject to extraction of RNA using bacterial RNA extraction kit (BIOMIGA, USA). Then, cDNA was obtained using first-strand cDNA synthesis kit (BIOMIGA, USA) according to the manufacturer’ instructions. The RT-PCR mixture consisted of 2 μl cDNA, 10 μl 2 × SYBR Green qPCR mix (Giagen, Beijing), 0.2 μM for each primer, and RNase-free water in a final volume of 20 μl. The PCR program was initiated at 95°C for 5 min, followed by 40 cycles at 95°C for 10 s, 59°C for 20 s, 72°C for 25 s using ABI Stepone plus (Applied Biosystems). The expression abundance of targeted genes between G362 and L3101 was determined in triplicate using ΔΔC_t_ method.

## Results And Discussion

**FIGURE 1:**
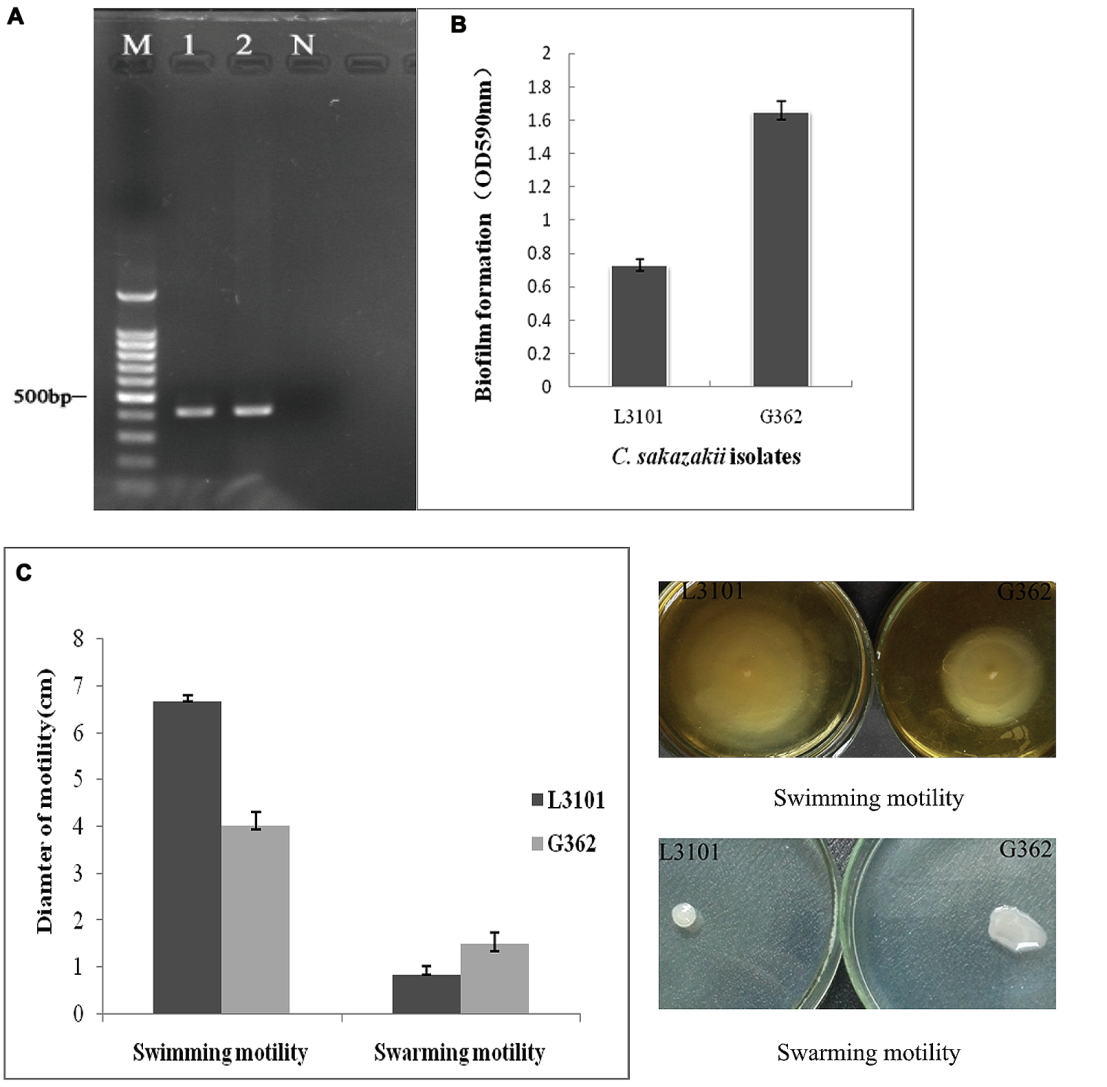
**The phenotypic characterization (O serotypes, biofilm formation, and motility) of *Cronobacter sakazakii* virulent G362 and attenuated L3101 isolate.** **(A)** Detection of O serotypes by PCR; **(B)** Biofilm formation using crystal violet staining (CVS); **(C)** Detection of swimming and swarming motility.

*Cronobacter sakazakii* is an important foodborne pathogen associated with severe infections in poor immunity or low-birth weight newborn. The virulence differences of *C. sakazakii* G362 and L3101were previously documented by Ye et al. (2015). In this study, the virulent characterization including biofilm formation, O serotypes, and motility was further determined. In **Figure 1A**, both G362 and L3101 were O5 serotype by PCR assay (Sun et al., 2012). Xu et al. (2014) found that O2 serotype in *C. sakazakii* is the predominant serotype among isolates from powdered milk. However, the correlation between O serotypes and virulence was not revealed. Additionally, the significant differences of biofilm formation between G362 and L3101 were observed in **Figure 1B**, which might be involved in their adhesion to surfaces. The biofilm formation was associated with virulence in *Candida* (Hasan et al., 2009), *Escherichia coli* (Naves et al., 2008), and *Pseudomonas* (Ghadaksaz et al., 2015). Interestingly, the differences of motility (swimming and swarming) in **Figure 1C** were also founded between G362 and L3101 at 37°C for 8 h (swimming motility) and 12 h (swarming motility). Swimming ability of L3101 is stronger than that of G362, while the weakest swarming motility was also observed in L3101. This is the first report to determine the relationship between flagella motility and biofilm formation in *C. sakazakii*. Our result preliminarily indicated that swimming motility had negative effects on the biofilm formation, while swarming motility contributed to the biofilm formation. The biofilm formation required flagella motility in *Pseudomonas aeruginosa* (Barken et al., 2008), and motility and adhesion to surface was also connected in *E. coli* (Wood et al., 2006). However, flagella motility was not necessarily required for biofilm formation in *P. aeruginosa* (Caiazza et al., 2007) and *Burkholderia pseudomallei* (Lee et al., 2010). In *C. sakazakii*, the regulation of biofilm formation and flagella motility remains to be revealed.

**FIGURE 2:**
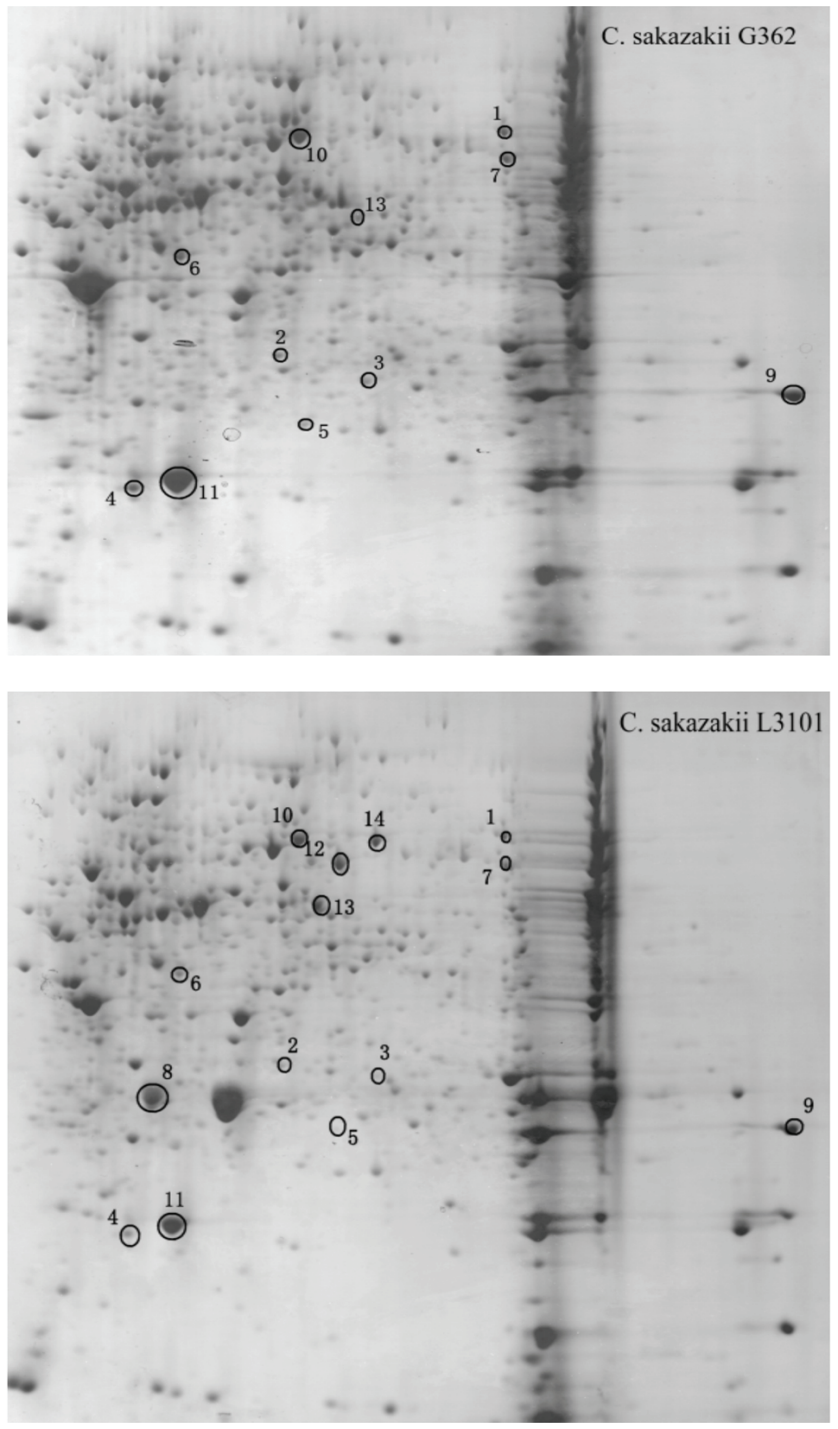
**Membranous proteomics profiles of virulent (G362) and attenuated (L3101) isolates.** pH 3-10 (from left to right).

**FIGURE 3:**
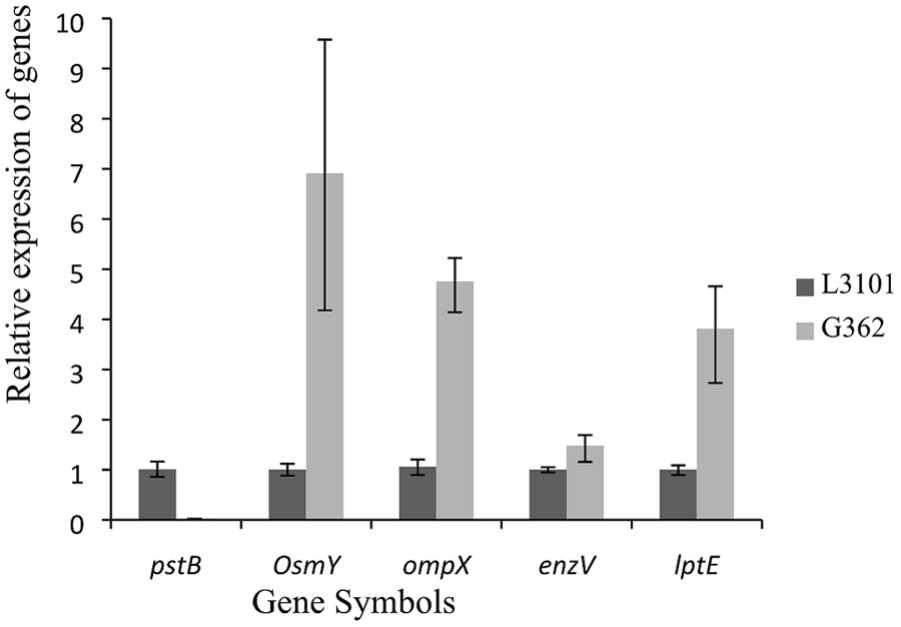
**Relative fluorescence quantitative PCR (RT-PCR) of membrane protein genes involved in virulence differences between G362 and L3101**.

Thereafter, to reveal the membranous proteins involved in virulence differences among *C. sakazakii* isolates, the membranous proteomic profiles of G362 and L3101 were firstly constructed using the 2-DE technology (**Figure 2**). Changes of membranous proteins in intensity (>twofold) were considered as significant differences. The expression abundance of 14 proteins was successfully identified between G362 and L3101. In **Table 1**, 8 out of 14 differentially expressed proteins was confirmed as membrane-associated proteins and expression abundance of seven membranous proteins was increased in G362. The different expression abundance of genes of membranous proteins including enzV, ompX, lptE, pstB, and OsmY were detected using real time PCR. The primers were listed in **Table 2** and 16S rRNA gene was used as internal control. From **Figure 3**, the expression of five membranous protein genes at mRNA levels using real-time PCR was consistent to results by 2-D method. The relative expression abundance of *osmF, ompX, enzV*, and *lptE* genes in G362 were 6.88, 4.49, 1.48, and 3.82 fold changes than those in L3101, respectively, while the expression of *pstB* in L3101 was 61.38 fold changes than that in G362. The relative expression of five factors by real-time PCR was consistent with results using 2-DE method.

**Table 1:** Identification and relative quantification of differentially expressed membranous proteins by MALDI-TOF/TOF MS for virulent *Cronobacter sakazakii* G362 compared to L3101.

Spot No.^a^	Identified protein	Mascot score^b^	UniProt No.^c^	Calculated pI^d^	Mass (Da)^e^	Sequence coverage	Matched peptide*^f^*	Protein localizaion	Increase or decrease (fold change)
(1)	Putative multidrug resistance protein MdtD	71	A7MHI9	10.18	50787.54	14%	8	Inner membrane	↑(2.04)
(2)	Osmolarity sensory histidine kinase EnvZ	48	K8D7W7	6.35	49995.71	8%	4	Inner membrane	↑(2.63)
(3)	LPS-assembly lipoprotein LptE	263	A7MQS1	6.91	20160.72	20%	8	Outer membrane	↑(2.01)
(4)	Outer membrane protein X	313	I2EKJ1	4.84	18244.18	43%	12	Outer membrane	↑(2.20)
(5)	Osmotically inducible protein OsmY	182	K8CMY3	6.76	21320.04	32%	7	Outer membrane	↑(2.06)
(6)	Outer membrane protein A	129	M1JW19	5.20	37080.54	22%	8	Outer membrane	↑(3.43)
(7)	Putative ABC transporter permease	89	I2EF46	9.21	34737.27	48%	5	Membrane	↑(2.49)
(8)	Phosphate import ATP-binding protein PstB	92	F5VNR7	6.24	29110.44	11%	3	Membrane	↓(Only in attenuated strains)
(9)	50S ribosomal protein L3	386	A7MPI7	9.87	22241.48	54%	8	Cytoplasmic	↑(2.67)
(10)	Putative maltose-6′-phosphate glucosidase	107	A7MQ03	5.75	49315.43	17%	5	Cytoplasmic	↑(2.18)
(11)	DNA protection during starvation protein	183	A7MEY6	5.49	18589.09	21%	4	Cytoplasmic	↑(2.33)
(12)	GDP-mannose 4,6-dehydratase	264	A7MHG3	5.84	42046.81	35%	12	Cytoplasmic	↓(Only in attenuated strains)
(13)	GDP-L-fucose synthase	114	A7MHG4	5.90	35896.77	25%	7	Cytoplasmic	↓(2.75)
(14)	Methylthioribose kinase	93	A7MKY0	5.83	44372.46	15%	9	Cytoplasmic	↓(Only in attenuated strains)


*^a^The number refer to the spot numbers as given in **Figure 1**.*

*^b^MOWSE scores of the highest confident matches (*P* < 0.05).*

*^c^Accession numbers in uniprot protein database (available at http://www.uniprot.org).*

*^d^pI, the predicted isoelectric point calculated from the protein sequence.*

*^e^The predicted molecular weight calculated from the protein sequence.*

*^f^Number of experimental peptides matched to the predicted protein peptides.*

**Table 2:** Primers used in the study by real-time PCR.

Genes	Sequences of primers (5′–3′)	size (bp)
*OmpX*	F:AAAAAGACCGCACTGAAGATGG	150
	R:TATCAGTGCCGTTGGTAGCCT	
*enzV*	F:TCCATCAGGGCGATTTCTC	150
	R:GAATAATGCCTTTACCGACCTG	
*LptE*	F:CCTCGGTCTTCCAGAACGGT	196
	R:GCGGCTTTGTCATACATCTCCT	
*OsmY*	F:GCTGAGCGGCTTTGTTGA	163
	R:GATTTCGCTGGTGGTGGC	
*pstB*	F:CGAAAACATTCTGAACCACTCC	197
	R:CGTTCCATAATGCGGCTTTG	
*16S*	F-ACGAGTGGCGGACGGTGA	238
*rRNA*	R-TCAGTTCCAGTGTGGCTGG	


Based on proteomic analysis, the outer membrane proteins (ompA and ompX) were increased in G362 compared to those in L3101. The ompA and ompX proteins were reported to enhance adherence and invasion of human cells (Mohan Nair and Venkitanarayanan, 2007; Mohan Nair et al., 2009; Liu et al., 2012). Outer membrane A of *E. coli* has been reported enhanced the invasion of brain microvascular endothelial (BMEC) by assisting through the blood-brain barrier (Prasadarao et al., 1996). Our findings also indicated that ompA and ompX might be involved in virulence differences of *C. sakazakii* at membranous protein levels.

Besides, the expression abundance of novel proteins of LPS-assembly lipoprotein LptE, Osmolarity sensory histidine kinase EnvZ, and Osmotically inducible protein OsmY in G362 were increased compared to those in L3101. The LPS-assembly lipoprotein LptE encoded by the gene *lptE* in *E. coli* is essential for the biosynthesis of lipopolysaccharide, resulting in strong immunogenicity with host cells (Chng et al., 2010). Inner membrane osmolarity sensory histidine kinase EnvZ-OmpR pair in *C. sakazakii* would be activated in response to environmental osmoic stress (Amalaradjou and Venkitanarayanan, 2011). The receptor ompR could influence bacterial biofilm formation and flagella motility by regulating genes *ompF* and *ompC*. Further findings indicated that virulence of *ompR* mutants decreased in *Salmonella typhimurium* (Bernardini et al., 1990; Raczkowska et al., 2011). Additionally, Osmotically inducible protein OsmY also played important roles in pathogenic bacteria. In *S. typhimurium* and *E. coli*, OsmY was indirectly associated with virulence behavior (Bader et al., 2003; Dong and Schellhorn, 2009). In addition, putative multidrug resistance protein MdtD and putative ABC transporter permease were also up-regulated in G362. Some authors reported mdtABCD together with BaeSR two-component system contributed to resisting to host defenses through membrane drug-eﬄux pump (Baranova and Nikaido, 2002; Zoetendal et al., 2008; Leblanc et al., 2011). ABC transporter permease, a multi-pass membrane protein, was responsible for oligogalacturonide transport, but its detailed mechanism involved in virulence of bacteria is not documented.

Interestingly, the expression abundance of some proteins like phosphate import ATP-binding protein PstB, GDP-L-fucose synthase and methylthioribose kinase were increased in L3101. To date, the correlation of these proteins and bacterial virulence was not documented.

In summary, the relationship between flagella motility and biofilm formation was preliminarily determined, which indicated that motility was not necessarily required for biofilm formation. Then, membranous proteomic profiles between virulent and attenuated *C. sakazakii* isolate were firstly constructed. In spite of ompA and ompX, some novel factors (envZ, LptE, MdtD, and OsmY) were successfully identified by proteomics which were potentially involved in virulence differences. The findings here provided novel insights and better knowledge for revealing pathogenic mechanisms of *C. sakazakii*. However, the detailed roles and regulation of these potential virulence factors of *C. sakazakii*, and study on interaction between host and bacteria are also urgent for exploration of pathogenic mechanism.

## Conflict of Interest Statement

The authors declare that the research was conducted in the absence of any commercial or financial relationships that could be construed as a potential conflict of interest.
